# Supplementary material for: Niche-Based Microbial Community Assemblage in Urban Transit Systems and the Influence of City Characteristics
Source: Microbiol Spectr. 2023 Mar 14;11(2):e00167-23. doi: 10.1128/spectrum.00167-23 (PMC10101094; doi:10.1128/spectrum.00167-23)
Supplement: Supplemental file 1 — Supplemental material. Download spectrum.00167-23-s0001.pdf, PDF file, 4.4 MB [file spectrum.00167-23-s0001.pdf]

Supporting Information

Niche-based microbial community profile and assemblage in urban transit systems and the influence of city characteristics

Guangzhou Xiong[1], Lei Ji[1], Mingyue Cheng[1], Kang Ning[1,*]

[1]Key Laboratory of Molecular Biophysics of the Ministry of Education, Hubei Key Laboratory of Bioinformatics and Molecular-imaging, Center of AI Biology, Department of Bioinformatics and Systems Biology, College of Life Science and Technology, Huazhong University of Science and Technology, Wuhan 430074, Hubei, China

[*]Corresponding authors:

Kang Ning, ningkang@hust.edu.cn

Ten pages are included, with two tables (S1 and S2) and six figures (Figures S1-S6) in this supporting PDF file.

Materials and Methods

Data configuration, model construction and validation

Three types of datasets, i.e., source dataset, transfer dataset, and query dataset, were used for mining city-specific microbes of different surface microbial communities (Figure S4). Take bench surface city-specific microbial mining, for example: bench surface samples were divided into the transfer dataset and query dataset. The transfer dataset contained 10 samples of each city that were randomly selected, and the query dataset contained the remaining samples (Figure S4). The source dataset contained the surface samples other than the collected bench surface samples. There was no sample overlap between the transfer dataset, query dataset, and source dataset (Figure S4).

To mine city-specific microbes on urban transit system surfaces, three kinds of random forest models were constructed using the source dataset and transfer dataset, i.e., base model (BM), independent model (IM), and transfer model (TM). The BM was trained on the source dataset; the IM was trained on the transfer dataset; and the TM was constructed by transfer from the BM using the transfer dataset (Figure S4). BM, IM, and TM were validated using query datasets (Figure S4). The data configuration, model training processes, and testing processes are presented and described in detail in Figure S4.

City characteristics, including the continent, latitude, longitude, population, population density, surface type, elevation, proximity to the coast, region, average June temperature and Koppen climate, were also recorded for each city. These city characteristics were used for establishment of the association between the microbial profiles and city characteristics.

Table S1. Sample counts by cities and surface types

Continent	City	Bench	Door	Handrail	Kiosk	Others	Total
East Asia	Hong Kong	0	68	179	0	465	712
	Seoul	21	0	0	0	59	80
	Singapore	0	0	35	14	137	186
Europe	Barcelona	0	0	15	16	93	124
	Kyiv	16	17	22	0	41	96
	London	183	0	176	0	172	531
	Marseille	0	0	28	24	60	112
	Zurich	34	0	0	41	4	79
Middle East	Doha	0	31	0	0	76	107
North America	Fairbanks	15	0	0	0	123	138
	New York City	63	28	33	53	479	656
Oceania	Sydney	0	0	18	0	44	62
South America	Ribeirao Preto	50	0	0	0	42	92
	Rio De Janeiro	0	0	0	21	124	145
	Santiago	0	0	12	0	14	26
Sub Saharan Africa	Ilorin	0	27	0	0	186	213
**Total**		382	171	518	169	2119	3359

Table S2. New York City sample counts by surface types

**Surface type**	**Number**
Bench	63
Ceiling Rail	11
Center Seat	13
Door	28
Handrail	33
Kiosk	53
Pole	15
Railing	17
Seat Rail	11
Sensor	20
Stairwell Railing	43
Ticket Machine	44
Turnstile	40
Vertical Pole	11
**Total**	402

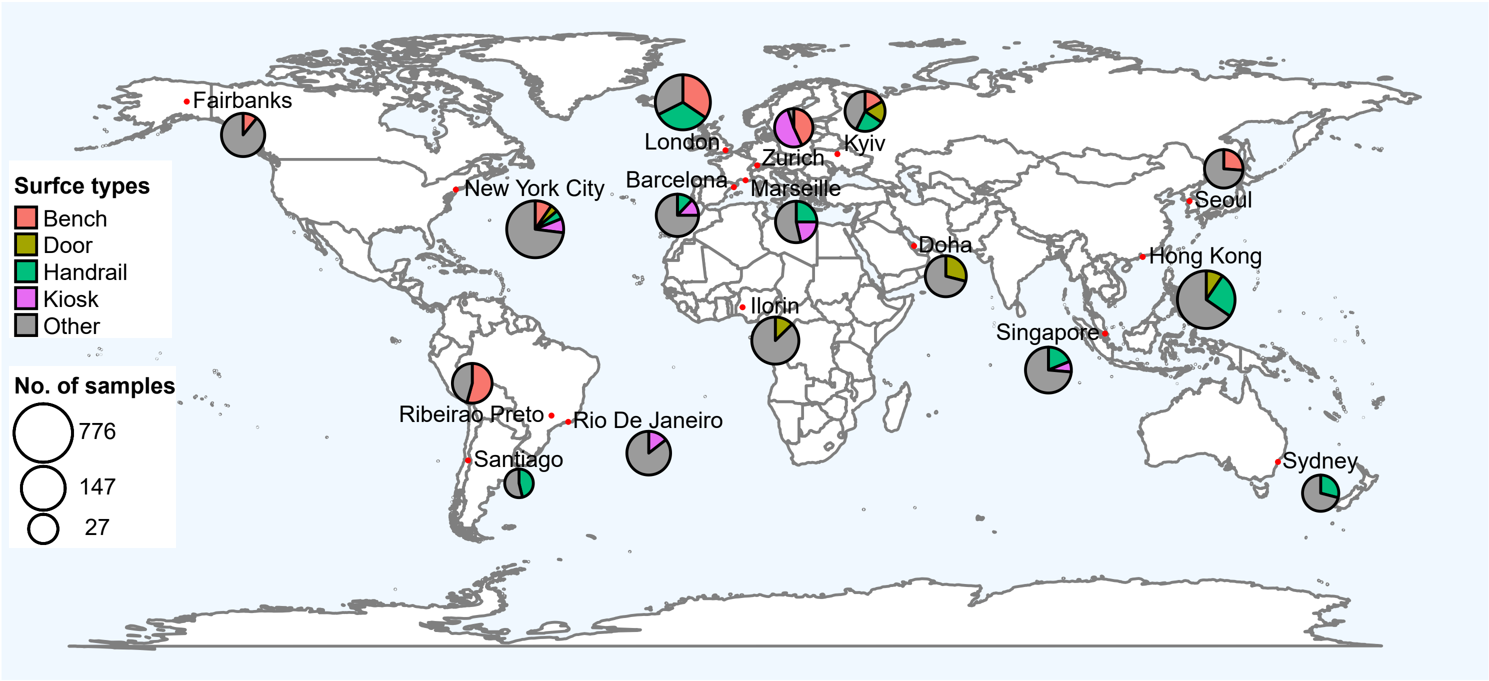

Figure S1. Global map showing the geographical distribution of all samples. The map shows the distribution of 3359 samples from 16 cities around the world. The color in the pie charts represents sample surface type. The size of the pie charts indicates the sample size.

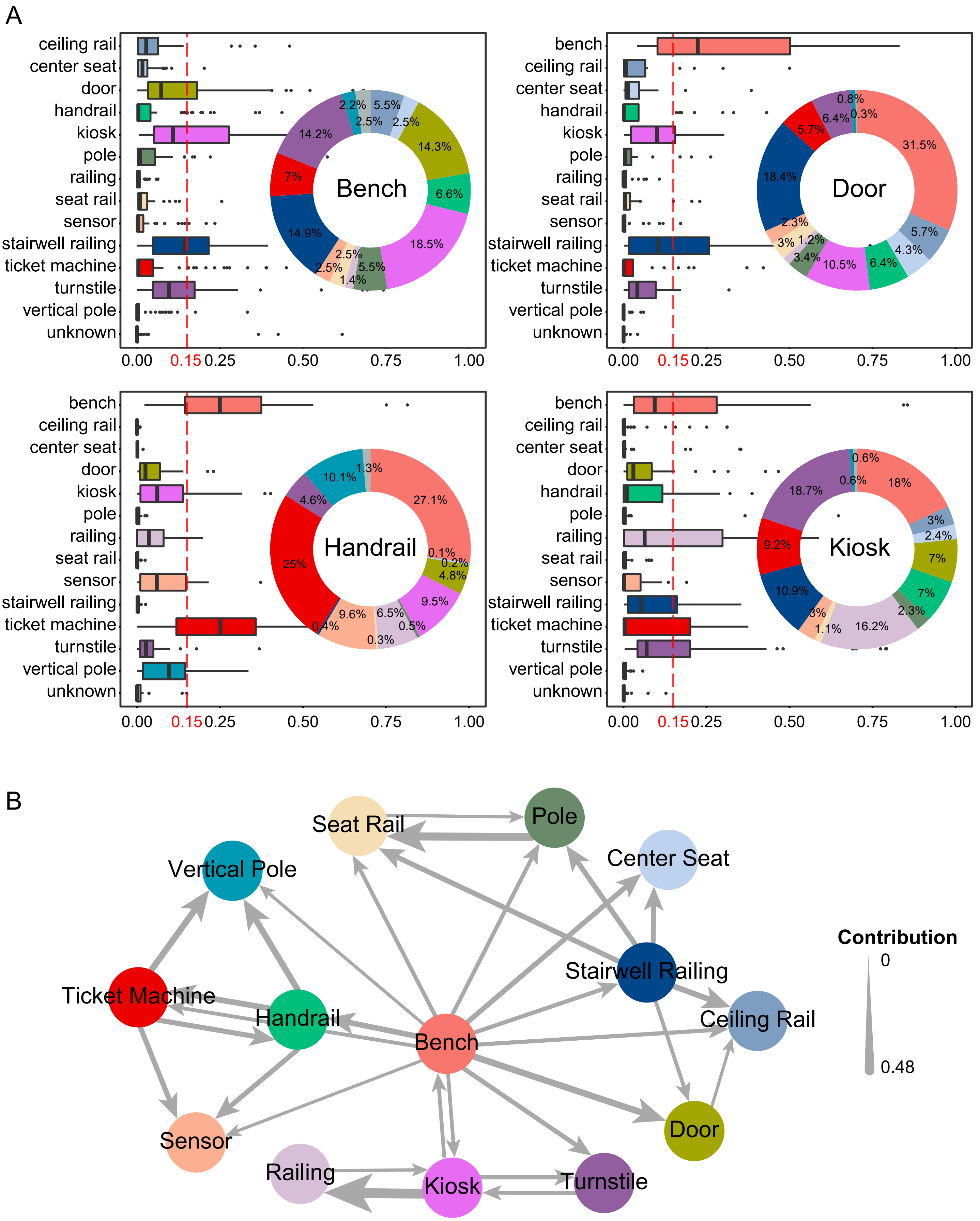

Figure S2. Associations between the New York City transit system different surface microbial communities. (A) The plots show different associations between the four main surfaces and other transit system surface microbial communities. Box-whisker plots show the association fractions' contribution (X-axis) between sink communities (Y-axis) when the four main surface microbiota was assigned as a sink, and the remaining samples were assigned as sources. The inset pie charts show the mean values of associations between the sink and the sources. (B) Network shows the source tracking contribution between different sample types in the New York transit system. Contribution values over 0.15 were chosen and shown. The contribution values from source sites to query sites were represented by the width of the edges.

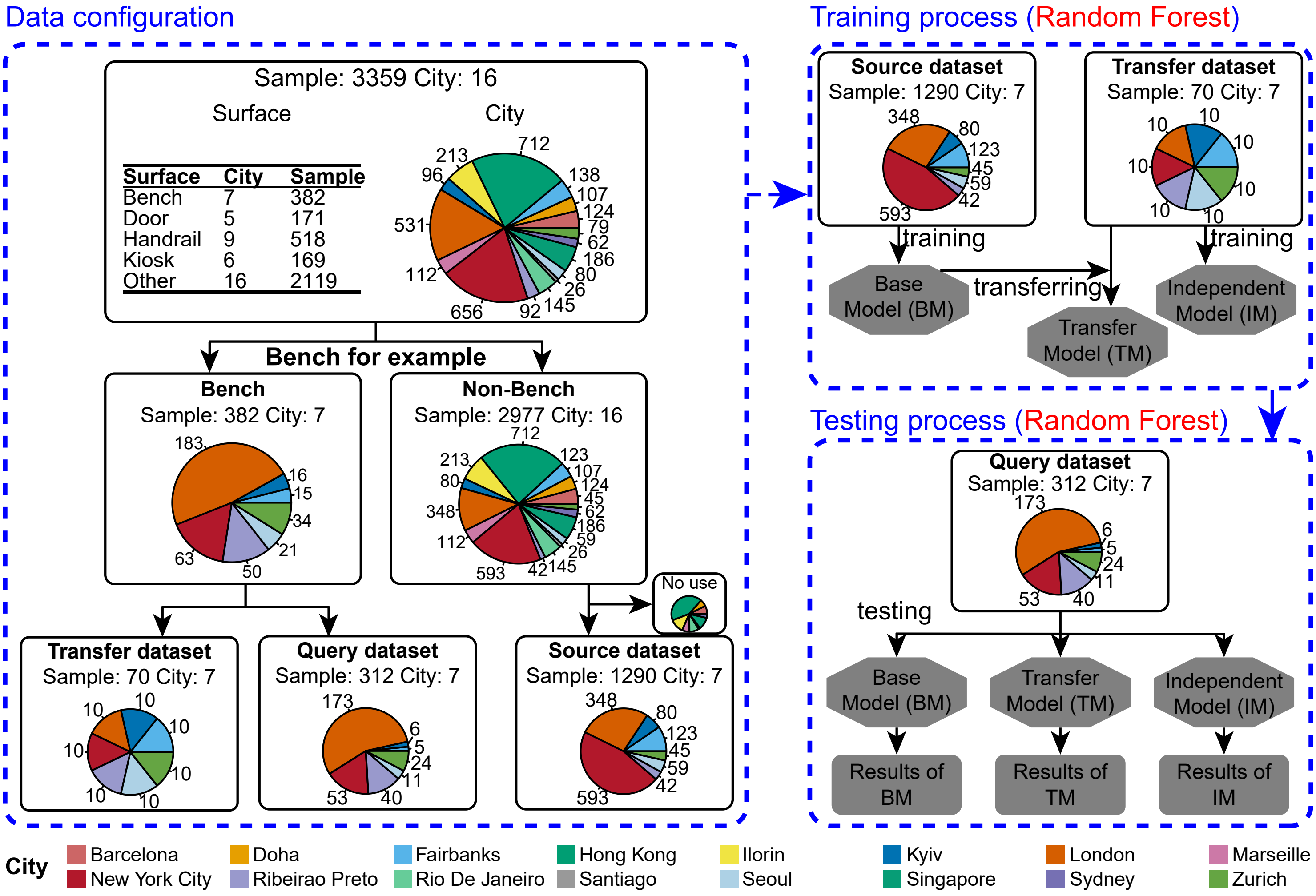

Figure S3. Schematic diagram of the microbial mining with random forest model-based transfer learning framework. In the data configuration process, 3359 urban surface samples, including four main surfaces (bench, door, handrail, and kiosk, N = 1240), were collected from 16 cities. Three types of datasets, i.e, source dataset, transfer dataset, and query dataset, were separated from all samples and used in mining city-specific microbes of different surface communities. Take bench surface city-specific microbial mining, for example: bench surface samples were divided into the transfer dataset and query dataset. The transfer dataset contained 10 samples of each city that were randomly selected, and the query dataset contained the remaining samples. The source dataset contained the surface samples other than the collected bench surface samples. There was no sample overlap between the transfer dataset, query dataset, and source dataset. Each color and number in the pie chart represents a city and the sample size for that city, respectively. In the model training process, three kinds of random forest models were constructed using the source dataset and transfer dataset, i.e., base model (BM), independent model (IM), and transfer model (TM). The BM was trained on the source dataset; the IM was trained on the transfer dataset; and the TM was constructed by transfer from the BM using the transfer dataset. In the model testing process, BM, IM, and TM were validated on query datasets. The data configuration, model training, and testing processes were repeated ten times.

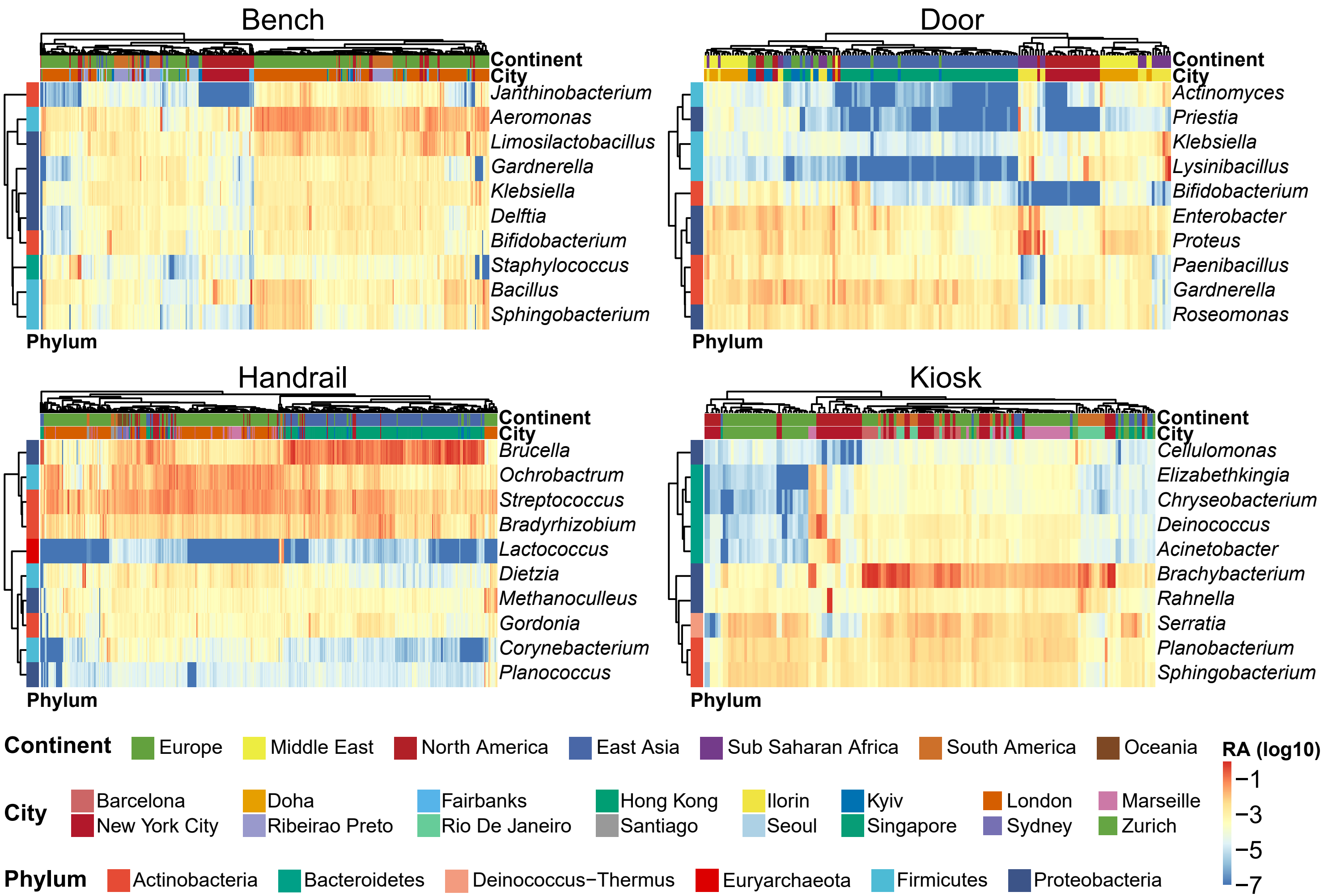

Figure S4. The city-specific microbes' geographical distribution pattern. The heatmap shows city-specific microbe (row), mind from bench, door, handrail, and kiosk surface communities, relative abundance in each sample (columns) across cities and continents. The color of the row represents the phylum of the city-specific microbes; The color of the column represents the city and continent of samples.

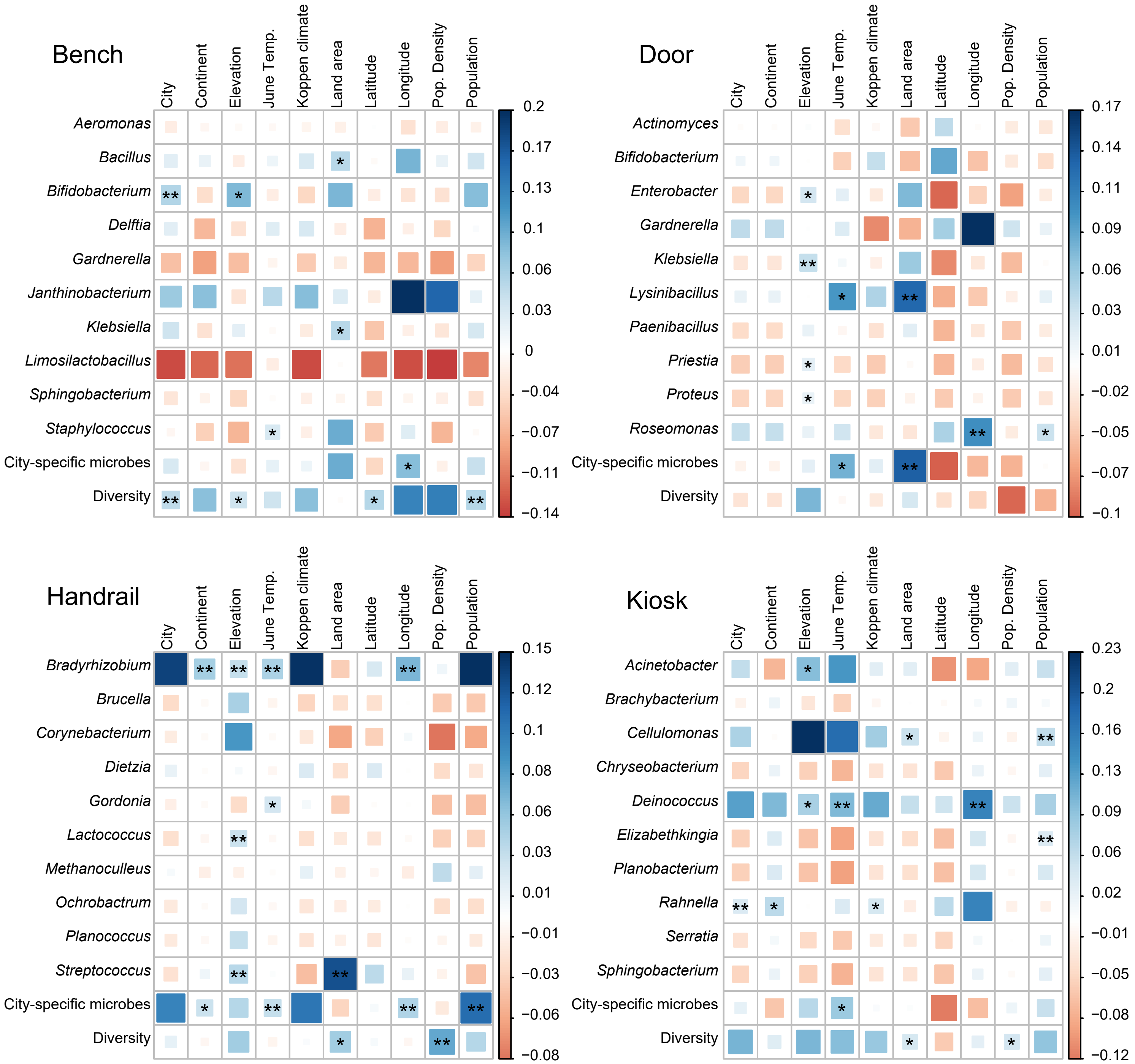

Figure S5. Correlations between the city-specific microbes and city characteristics and the correlations between community diversity and city characteristics in each main surface types. The heatmap shows the correlation between city-specific microbe relative abundance (rows) and city characteristics (columns) and the correlation between urban transit system surface community diversity (rows), and city characteristics (columns). The correlation coefficient was calculated by Mantel test. The city's characteristics include the city, continent, elevation, average June temperature, Koppen climate, region, latitude, longitude, population density, and population. City-specific microbes in heatmap row means the relative abundance. Diversity in heatmap row means the Shannon diversity index of the microbial community. Abbreviations: "**": $p < 0.01$; "*": $0.01 \le p < 0.05$;

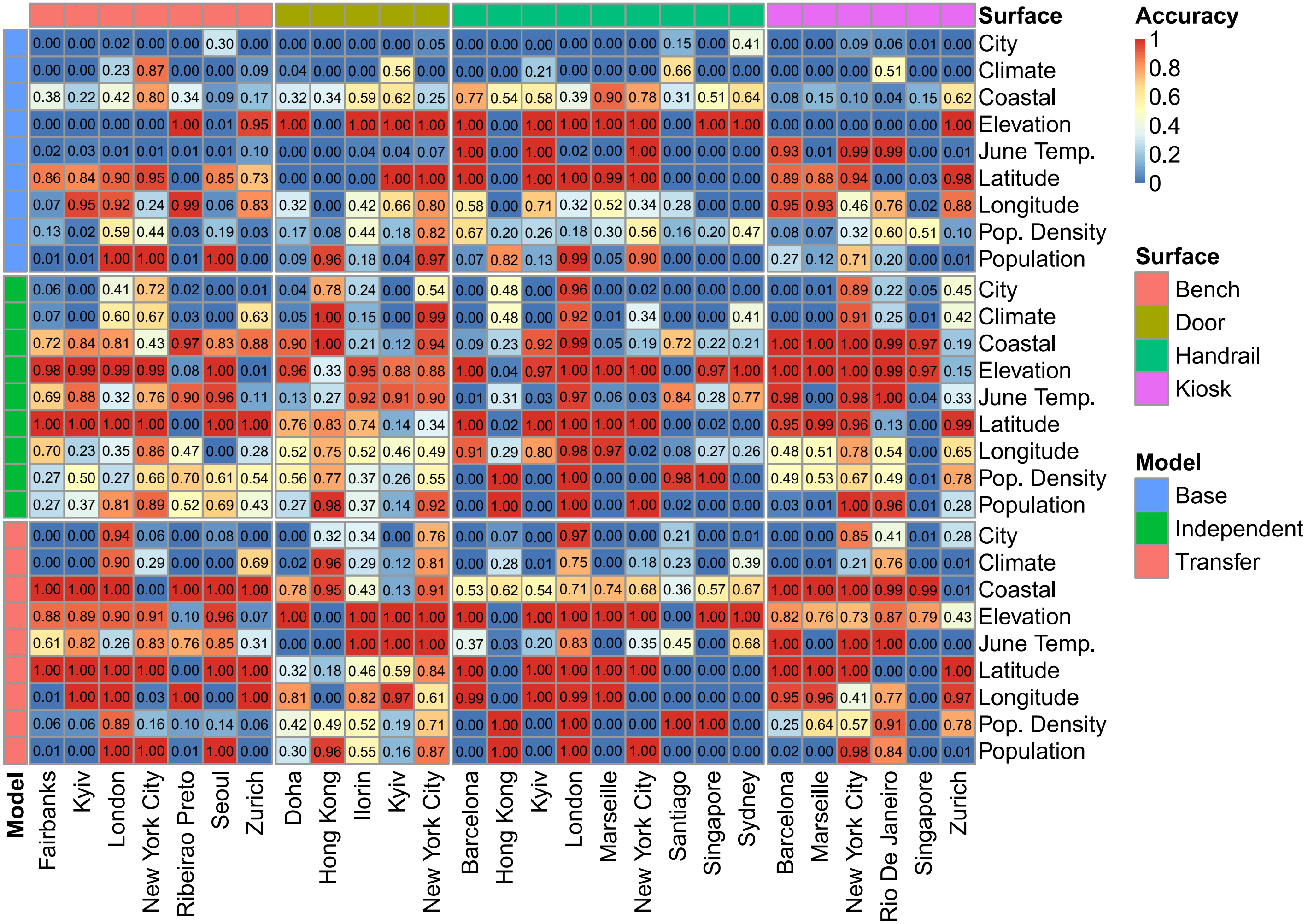

Figure S6. The performance of city characteristic classification models using the non-city specific genera as classification features. The heatmap shows the mean classification accuracy of the random forest model, using all genera except 50 most important city-specific microbes as classification features, for a given feature (rows, including the city, Koppen climate, proximity to the coast, elevation, average June temperature, latitude, longitude, population density, and population) in samples from a city (columns) on four main surface datasets respectively. The color of the row represents the type of prediction model; The color of the column represents the surface type which city-specific microbes that were used in the model training process mined from. Each main surface set was randomly divided into the training set and testing set (80%:20%). The base model was trained using the other three main surface data. The data configuration, model training processes, and testing processes were repeated ten times. Continuous features (e.g., population) were discretized.
